# AI-accelerated protein-ligand docking for SARS-CoV-2 is 100-fold faster with no significant change in detection

**DOI:** 10.1038/s41598-023-28785-9

**Published:** 2023-02-06

**Authors:** Austin Clyde, Xuefeng Liu, Thomas Brettin, Hyunseung Yoo, Alexander Partin, Yadu Babuji, Ben Blaiszik, Jamaludin Mohd-Yusof, Andre Merzky, Matteo Turilli, Shantenu Jha, Arvind Ramanathan, Rick Stevens

**Affiliations:** 1 Argonne National Laboratory, Data Science and Learning Division, Chicago, Lemont 60439 USA; 2 Department of Computer Science, University of Chicago, Chicago, 60637 USA; 3 Department of Electrical and Computer Engineering, Rutgers University, Piscataway, 08854 USA; 4 Brookhaven National Laboratory, Computational Sciences Initiative, Upton, 11973 USA; 5 Los Alamos National Laboratory, Computer, Computational, and Statistical Sciences, Los Alamos, 87545 USA; 6 Argonne National Laboratory, Computing, Environment, and Life Sciences Directorate, Lemont, 60439 USA; 7 University of Chicago, Globus, Chicago, 60637 USA

**Keywords:** Computer science, Scientific data, Computational biology and bioinformatics, Developmental biology

## Abstract

Protein-ligand docking is a computational method for identifying drug leads. The method is capable of narrowing a vast library of compounds down to a tractable size for downstream simulation or experimental testing and is widely used in drug discovery. While there has been progress in accelerating scoring of compounds with artificial intelligence, few works have bridged these successes back to the virtual screening community in terms of utility and forward-looking development. We demonstrate the power of high-speed ML models by scoring 1 billion molecules in under a day (50 k predictions per GPU seconds). We showcase a workflow for docking utilizing surrogate AI-based models as a pre-filter to a standard docking workflow. Our workflow is ten times faster at screening a library of compounds than the standard technique, with an error rate less than 0.01% of detecting the underlying best scoring 0.1% of compounds. Our analysis of the speedup explains that another order of magnitude speedup must come from model accuracy rather than computing speed. In order to drive another order of magnitude of acceleration, we share a benchmark dataset consisting of 200 million 3D complex structures and 2D structure scores across a consistent set of 13 million “in-stock” molecules over 15 receptors, or binding sites, across the SARS-CoV-2 proteome. We believe this is strong evidence for the community to begin focusing on improving the accuracy of surrogate models to improve the ability to screen massive compound libraries 100 × or even 1000 × faster than current techniques and reduce missing top hits. The technique outlined aims to be a fast drop-in replacement for docking for screening billion-scale molecular libraries.

## Introduction

Viral pandemics, antibiotic-resistant bacteria, or fungal infections such as *Candida auris* are fundamental threats to human health^1,2^. SARS-CoV-2 (COVID-19) shocked the world with its first appearance estimated in the Fall/Winter of 2019 to becoming a global crisis by March 2020 when it was declared a global pandemic by the World Health Organization. Even with the rapid development of vaccines, molecular therapies remain a critical tool for reducing mortality and morbidity^3^. The development of a small molecule inhibitor of the virus is an important tool yet to come into fruition. We believe the ML community can aid in an effort for global preparedness by developing computational infrastructure to scale computational drug discovery efforts. High throughput computational techniques can be used at the beginning of pandemics or even as surveillance systems by screening billions of compounds against entire proteomes to find the most promising leads.

In response to the COVID-19 pandemic, scientists across the globe began a massive drug discovery effort spanning traditional targeted combinatorial library screening^4–8^, drug repurposing screens^9,10^, and crowd-sourced community screening^11^. Programs such as the JEDI COVID-19 grand challenge aimed to screen over a billion molecules. In common to all these efforts was the ability to leverage off-the-shelf molecular docking programs rapidly. Molecular docking programs are essential to preliminary drug discovery efforts as they predict the 3D structure of drug candidates in complex with the protein targets of focus.

**Figure 1:**
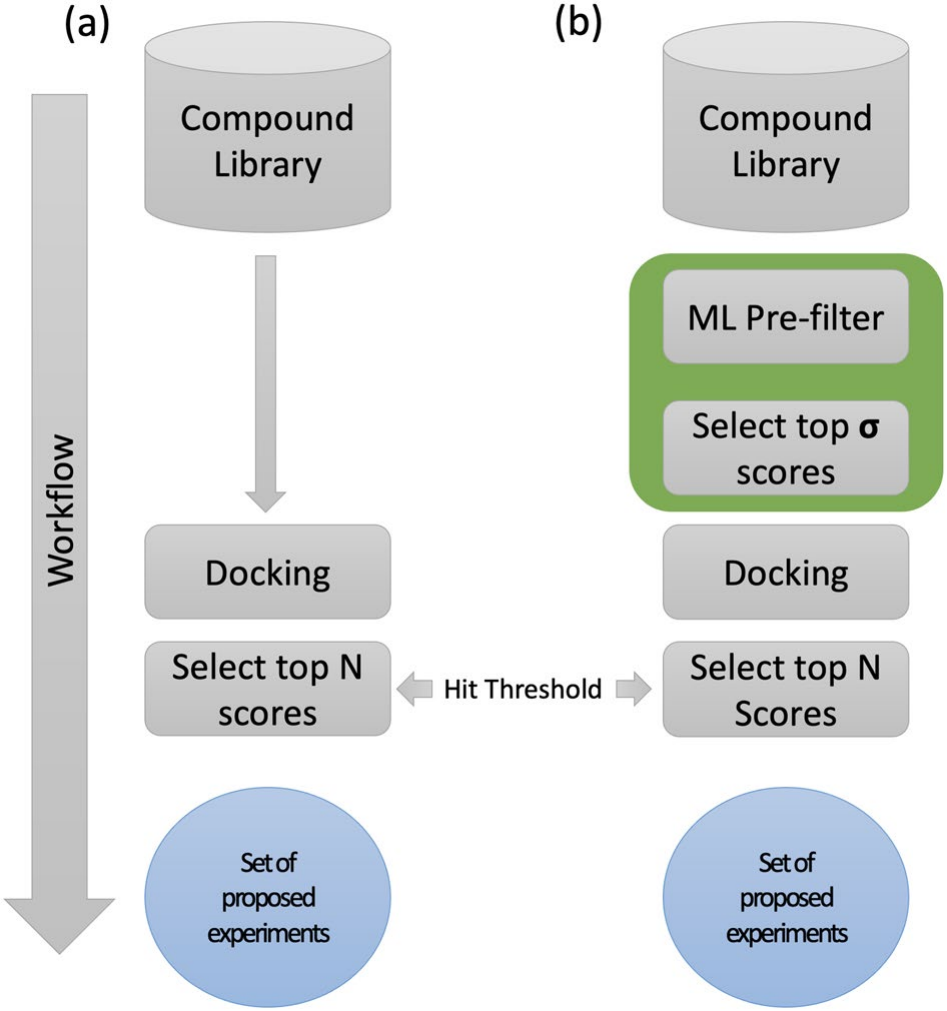
Overview of surrogate prefilter then dock. (**a**) This workflow is the standard virtual screening workflow consisting of taking a compound library, docking the library, and selecting the top scoring compounds. (**b**) This workflow is the surrogate prefilter and dock (SPFD) workflow where there are two thresholds, both the hit threshold as in (**a**) but also the $\sigma$ threshold which decides how many compounds to pass through to docking. Ultimately, in order to not miss the few leads that would normally pass to experiments, our evaluation technique aims to ensure that the set of experiments out of both workflows matches.

The first phase of computational drug discovery often starts with identifying regions of a protein (receptors) that are reasonable targets for small molecule binding, followed by searching small molecules for their ability to bind the receptor. These receptor-binding computations are performed using standard docking software, such as AutoDock ^12–14^, UCSF Dock ^15,16^, and many others^17^. These molecular docking programs search the conformational and positional space of the ligand with respect to the receptor until a scoring function is minimized, resulting in an affinity score. An affinity score is then used to rank candidate poses. A cutoff is applied along with post-processing^18,19^, and the resulting set is passed along for downstream study (Fig. 1a). Behind this workflow is protein-ligand docking software, which has two main outputs: a pose (ligand conformer in a particular complex with the protein) and an associated score. Scores are approximated to the binding free energy, though the units and interpretation of scores depend on the exact docking protocol used. The inputs are an ensemble of 3D ligand conformations and a protein receptor. We call a protein receptor a protein structure that has been annotated with particular binding pocket coordinates (i.e. the binding site box).

There are several open databases of molecules commonly used for virtual screening. These compound databases come in different varieties such as commercially available compounds, theoretically synthesizable compounds, and compounds that have an unknown synthesizability. Some of the largest include ZINC^20^, Enamine Real^21^, GDB-13^22^, and SAVI^23^ with each containing 10^9^ or more compounds. Recently, Babuji et al. released an aggregate collection of over 10^9^ compounds in representations suitable for deep learning^24^. These representations included drug descriptors, fingerprints, images, and canonical smiles. Searching collections of this size using traditional docking tools is not practical as even a single target screening takes many days of supercomputing time^25^.

Several accelerated docking protocols have been studied. Progressive docking utilizes subsets of a compound library’s docking results to build predictive models for the remaining library resulting in a speed up of 1.2 to 2.6 fold over traditional full library docking^26^. Spresso compresses compound libraries based on similar fragments reducing the library by over 200 times resulting in faster docking^27^. Virtual flow utilizes fast molecular docking (such as AutoDock Vina or QuickVina 2) in conjugation with conformational sampling in a staged system with high-accuracy docking to screen large chemical libraries^28^. Deep docking bootstraps a deep learning model on a subset of a compound library and then utilizes the model to pick off top scoring molecules^29^.

Lean docking uses a regressor trained on 10 k docked ligands^30^. Validation on the LIT PCBA^31^ dataset have shown that lean docking can accelerate screening between 4 to 41 times (depending on docking screening performance on a given protein target) without loss of top-scoring true actives.

This work demonstrates the application of deep learning to accelerate docking, thereby expanding our ability to search more extensive libraries of small molecules. We do so by first illustrating our large-scale effort of training surrogate models across the SARS-CoV-2 proteome. We demonstrate our method of virtual ligand screening called Surrogate Prefilter then Dock (SPFD) (Fig. 1b). SPFD utilizes an ML-based surrogate docking model to prefilter a compound library and then uses classical docking to locate the final hit set from the ML-enriched prefiltered set. The SPFD method contextualizes the application of ML models to virtual screening by utilizing the ML model as a pre-screening filter to reduce the library to a tractable size for standard virtual ligand screening. After pre-filtering, a standard docking protocol is applied. In practice, this separates the ML from the typical workflow, which the virtual screening community has well studied^32^. We perform a detailed analysis of SPFD by combining model accuracy performance, computational throughput, and virtual screening detection sensitive into a single analysis framework. Our analysis framework shows that pre-screening with an ML surrogate model is 10x as fast as the traditional only docking method with less than 0.1% error of detecting top hits. In practice, this means SPFD can reduce the time to screen a compound library by a factor of ten without losing any potential hits one may have found if one ran standard virtual ligand screening on the whole original library. What is unique to our framework is that we analytically show that the ability to improve this speedup factor beyond a single order of magnitude is limited by surrogate model accuracy rather than computing power. We hope that releasing the most extensive docking dataset with sequential, 2D and 3D formulations and our benchmarks models will allow the community to improve the modeling aspect, leading to tangible 100x or even 1000x improvement in throughput for structure-based virtual screening. As hardware accelerators for ML models continue to grow our ability to run inferences orders of magnitude faster rapidly, we must push on understanding how to advance detection accuracy for ML-based virtual screening models.

## Related work

Virtual screening is a broad category of computational techniques for searching databases of compounds to locate an exciting subset of leads for downstream tasks^33^. The goal of virtual screening is to propose molecules for testing in biological assays. Generally, the space of compounds available is many orders of magnitude beyond what is feasible for wet-lab testing. Standard practice in virtual screening is to utilize molecular docking. Molecular docking is a computational technique for evaluating the various poses a ligand can take in a protein binding pocket^34^.

From an algorithmic perspective, molecular docking is a computational means of assigning a favorability score to a molecule for a particular protein pocket. Various groups work with molecular docking at scale to discover new chemotypes or potent therapeutics^25,35,36^. Recently, more groups have begun to develop machine learning models re-score the scores coming from docking (i.e., connecting the computational scores from a model of energetics to success in in-vitro assays)^37,38^. Other groups have tried to address the computational throughput problem of docking^39,40^. Researchers have attempted to redesign docking protocols to run on GPUs or to reduce the complexity of docking through further approximations^41–43^.

In general, we have found the adoption of ML techniques to be a difficult task in the biological sciences^44^. To the best of our knowledge, no benchmark or public dataset release focuses on bridging the gap between the virtual screening community and the machine learning community. In this paper, the benchmark we outline attempts to bridge this gap. We propose utilizing surrogate models as a pre-filter before docking. We believe this will increase the potential adoption of ML in biological research as (1) it reduces any epistemic reliance on the surrogate models and (2) directly addresses the communities’ current problem of interest, which is expanding chemical library size to go beyond the standard molecule libraries which have been screened time and time again for the past 30 years^45^. Our proposal for surrogate models as a pre-filter, SPFD, solves an epistemic problem since the ML model is designed to filter a sizeable molecular library down to a computationally tractable library size. The virtual screening community can continue their standard practice of docking on (or whichever downstream protocol they desire). SPFD positions any gains from the ML research community as a starting point for the drug discovery community rather than a middle-man where epistemic reliance may be required. Second, our benchmark proposal focuses directly on expanding the tractability of computing large library sizes, which has been an impetus in drug discovery. Together, we believe this situates our benchmark apart from currently published benchmarks and works towards a fundamental problem of building bridges between communities. Furthermore, this motivates a novel mode of statistical thinking for ML researchers. Instead of focusing on the central tendency of the dataset, this regression context of predicting computational scores requires studying how to detect tiny subsets of a library (0.01% or less) with near-perfect precision.

## Dataset overview

As part of our drug discovery campaign for SARS-CoV-2^6^ , we developed a database of docked protein-ligands across 15 protein targets and 12M compounds as well as the complexes’ associated scores. The data preparation is outlined in the prior work. In brief, ligands were prepared using OpenEye Scientific OMEGA toolkit where 300-900 conformations were sampled for each ligand^46^. Receptors were prepared using the OEDOCK application. If the active site was unknown at the time, FPocket was used and the three highest scoring binding sites were used as an ensemble^47^.

The database contains two related tasks. The first task is predicting a ligand’s docking score to a receptor based on 2D structural information from the ligand. The second possible task is a pan-receptor model that encodes the protein target to use a single model across different ligands and targets. These tasks are distinct from other drug discovery datasets as this benchmark is focused directly on surrogate model performance over the baseline computational drug discovery method of docking. A different approach to applying machine learning to docking is the use ML models as a scoring function rather than the result of the optimization of the ligand conformation/position relative to the scoring function^48^. Other benchmarks are available to address to the gap between docking, and experimental binding free energy calculations such as DUD-E^49^.

The dataset we are releasing has three modes of representation, sequential, 2D or 3D, where the 3D data are a ligand conformation in an SDF file. 2D ligand data are available in a CSV file containing the molecule’s purchasable name, a SMILES string, and its associated docking score in a particular complex.

The sequential dataframe includes maccs-key^50^, ecfp2^51^ , ecfp4, ecfp6 fingerprints, and descriptors. We provided baselines and feature descriptions in Appendix 0.1 . The models discussed in the rest of the main paper pertain to the 2D ligand structures (the associated 3D data are shared with the community for further developing 3D modeling techniques^52^) .

The ligands available for each dataset are sorted into three categories ORD (orderable compounds from Mcule^53^), ORZ (orderable compounds from Zinc^20^), and an aggregate collection which contains all the available compounds plus others (Drug Bank^54^, and Enamine Hit Locator Library^55^). Docking failures were treated as omissions in the data, which may be important consideration though typically, the number of omissions accounts for 1–2% at most of each sample.

The data are available here, https://doi.org/10.26311/BFKY-EX6P, and more information regarding persistence and usage is available on the data website^56^. An exciting extension work based on this dataset could be the ensemble, multi-modal, or active model selection methods^57,58^, which utilize multiple features of this dataset.

## Methods

At a high level, surrogate models for protein-ligand docking aim to accelerate virtual ligand screening campaigns. A surrogate model seeks to replace the CPU-bound docking program with a trained model. In this case, surrogate models alone are not a viable solution to protein-ligand docking in general. ML surrogate models are based on gaussian statistics and generally perform well on predicting the central tendency of data, but not so at picking out the finer top or bottom 1%. We propose utilizing the ML to filter incoming ligands utilizing SPFD. Thus, the number of actual docking calculations is minimized compared to the typical approach of docking the entire dataset. Due to model accuracy, the number of missed compounds is minimized as the fine-grained selection of a hit set comes from traditional docking and the model only needs to select a coarse set of hits rather than a fine set. In other words, a surrogate model is trained, and a cut-off is specific, say 1%. The model is run over the proposed library to screen, and the top 1% of ligands are then docked utilizing the program to have the exact scores and pose information as with typical docking. In this way, we do not see current surrogate models as a replacement for docking but rather as a mean of expanding their use over large virtual libraries. This model has a single hyperparameter, $\sigma$, which determines after running the surrogate model over the library which percentage of most promising predicted compounds we then dock utilizing traditional docking techniques.

### Docking pipeline

The training and testing datasets for these experiments were generated using 31 protein receptors, covering 9 diverse SARS-CoV-2 viral target protein conformations, that target (1) 3CLPro (main protease, part of the non-structural protein/ NSP-3), (2) papain like protease (PLPro), (3) SARS macrodomain (also referred to as ADP-ribosyltransferase, ADRP), (4) helicase (NSP13), (5) NSP15 (endoribonuclease), (6) RNA dependent RNA polymerase (RDRP, NSP7-8-12 complex), and (7) methyltransferase (NSP10-16 complex). For each of these protein targets, we identified a diverse set of binding sites along the protein interfaces using two strategies: for proteins that had already available structures with bound ligands, we utilized the X-ray crystallographic data to identify where ligand densities are found and defined a pocket bound by a rectangular box surrounding that area; and for proteins that did not have ligands bound to them, we used the FPocket toolkit that allowed us to define a variety of potential binding regions (including protein interfaces) around which we could define a rectangular box. This process allowed us to expand the potential binding sites to include over 90 unique regions for these target proteins. We use the term target to refer to one binding site. The protocol code can be found here: https://github.com/2019-ncovgroup/HTDockingDataInstructions.

Two ligand libraries were prepared. The first was the orderable subset of the Zinc15 database (we refer to this as OZD) and the second was the orderable subset of the MCULE compound database (we refer to this as ORD)^6^. The generation of the orderable subsets was primarily a manual activity that involved finding all compounds that are either in stock or available to ship in three weeks across a range of suppliers. These are included in the set of molecular libraries examined in this study (SI Table 3). Consistent SMILE strings and drug descriptors for the orderable subsets of the Zinc15 and MCULE compound databases were generated as described by Babuji et al.^24^. Drug descriptors for the Zinc15 and MCULE compound databases can be downloaded from the nCOV Group Data Repository at https://2019-ncovgroup.github.io.

### Data frame construction

We used the protein-ligand docking results between the prepared receptors and compounds in the OZD library to build machine learning (ML) data-frames for each binding site. The raw docking scores (the minimum Chemgauss4 score over the ensemble of conformers in a ligand-receptor docking simulation) were processed^59^ . Because we were interested in determining strong binding molecules (low scores), we clipped all positive values to zero. Then, since we used the ReLu activation function at the output layer of the deep neural network, we transformed the values to positive by taking the absolute value of the scores. The processed docking scores for each compound to each binding site then served as the prediction target. The code for model training can be found here: https://github.com/2019-ncovgroup/ML-Code.

The features used to train the models were computed molecular descriptors. The molecular descriptors were computed as described by Babuji et al.^24^. The full set of molecular features is derived from the 2D ligand structures. The molecular features consist of 2-D and 3-D descriptors where 3D-descriptors are computed from the 2D structure using high-performance kernels^60^ . The feature set results in a total of 1,826 descriptors. The approximately 6 million docking scores per receptor and 1,826 descriptors were then joined into a data frame for each receptor.

### Learning curves

We performed learning curve analysis with the 3CLPro receptor to determine the training behavior of the model^61^. A subset of 2 M samples were obtained from the full set of 6 M samples. The 2 M sample dataset was split into train (0.8), validation (0.1), and test (0.1) sets. We trained the deep neural network on subsets of training samples, starting from 100 K and linearly increasing to 1.6M samples (i.e., 80% of the full 2 M set). Each model was trained from scratch and we used the validation set to trigger early stopping and the test to calculate measures of generalization performance such as the mean absolute error of predictions.

### Model details

The model was a fully connected deep neural network with four hidden layers (with neuron counts [250, 125, 60, 30, 1]), with dropout layers in between. The dropout rate was set to 0.1. Layer activation was done using the rectified linear unit activation function. The number of samples per gradient update (batch size) was set to 32. The model was compiled using mean squared error as the loss function and stochastic gradient descent (SGD) with an initial learning rate of 0.0001 and momentum set to 0.9 as the optimizer. The implementation was python using Keras^62^.

The model was trained by setting the initial number of epochs to 400. A learning rate scheduler monitored the validation loss and reduced the learning rate when learning stagnated. The number of epochs with no improvement after which the learning rate was reduced (patience) was set to 20. The factor by which the learning rate will be reduced was set to 0.75, and the minimum allowable learning rate was set to $10^{-9}$. Early stopping was used to terminate training if after 100 epochs the validation loss did not improve.

Features were standardized by removing the mean and scaling to unit variance before the onset of training using the entire data frame (before the data frame was split into train and test partitions). The train and test partitions were based on a random 80:20 split of the input data frame. Hyperparameter optimization was performed.

Inferencing was performed on Summit. The input was converted to Feather files using the python package feather, a wrapper around pyarrow.feather (see the Apache Arrow project at apache.org). Feather formatted files as input in our experience are read faster from disk than parquet, pickle, and comma-separated value formats.

## Results

### Identification of protein targets and binding receptors

**Figure 2:**
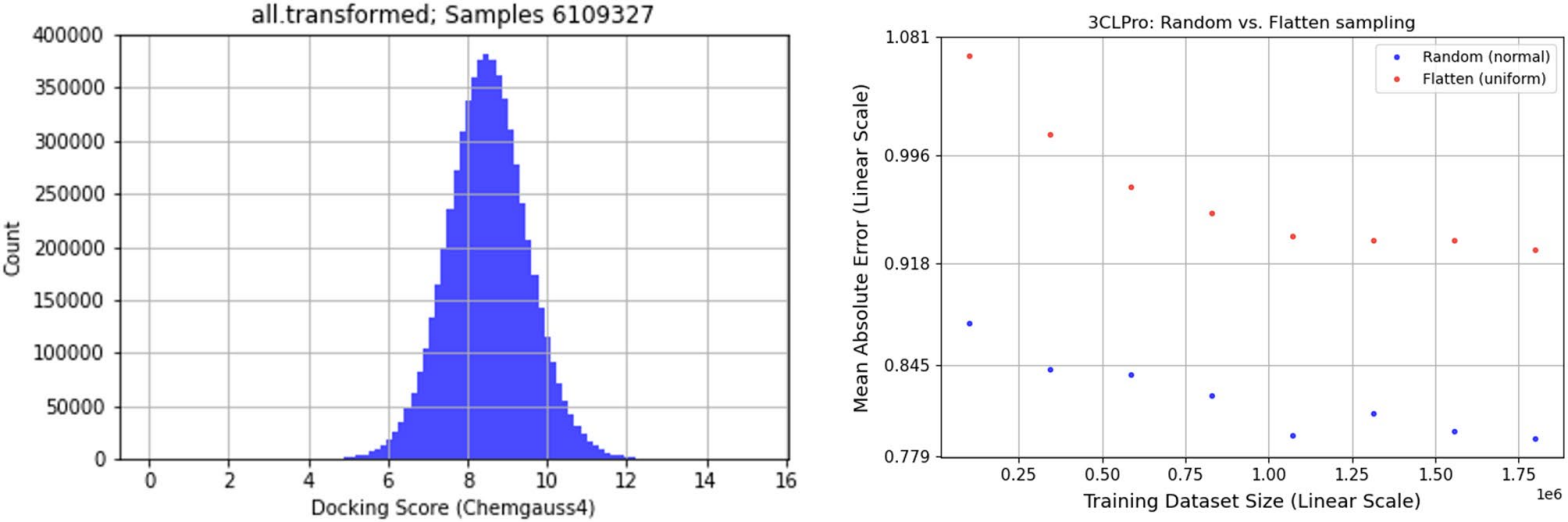
(left) Histogram of protein-ligand docking of transformed docking scores for 3CL-M$_{\textrm{pro}}$. The distribution is from the ORZ dataset based on the transformed 2D scores. (right) Learning curve between dataset size and MAE between random and flattened datasets.

A total of thirty one receptors representing 9 SARS-CoV-2 protein conformations were prepared for docking^56^. These are illustrated in Fig. 2 and listed in Table 1. The quality of the receptors reflect what was available at the time the receptor was prepared. For example, whereas the NSP13 (helicase) structure in Table 1 was based on homology modeling, today there exists X-ray diffraction models.

### Generation of training data

The results for the 3CLPro receptor demonstrate a normal distribution (Fig. 2). The best docking scores would be in the range of 12 to 18. The distribution of docking scores for the 3CLPro receptor is illustrative of the distributions for all the other receptors. As shown in the figure, there are very few samples with good docking scores relative to the entire set of samples.

### Sampling comparisons

We constructed a set of data frames to investigate the impact of the number of samples, sampling approach, and the choice of drug descriptors as features. The number of samples was further investigated using learning curves. Because we are interested in predicting docking scores in the tail of the distribution where the best docking scores exist, we explored two sampling approaches. Lastly, we investigated the impact of using the Mordred 3-D descriptors as features of the compounds.

**Table 1:** The four sampling approaches used to subset the approx.

Dataset	Count (samples)	Sampling method	Distribution (approximate)
100 K-random	100,000	Random	Normal
100 K-flatten	100,000	Flatten	Uniform
1 M-random	1,000,000	Random	Normal
1 M-flatten	1,000,000	Flatten	Uniform

6M docking scores for OZD.

We generated a dataset subset by sampling the approximately 6M samples in the OZD data complete data-frames. We examined four sampling approaches, differing by two parameters, as listed in Table 1: (1) the total number of samples drawn from the entire dataset (i.e., the count), and (2) the algorithm used to draw the samples (i.e., the sampling method).

**Figure 3:**
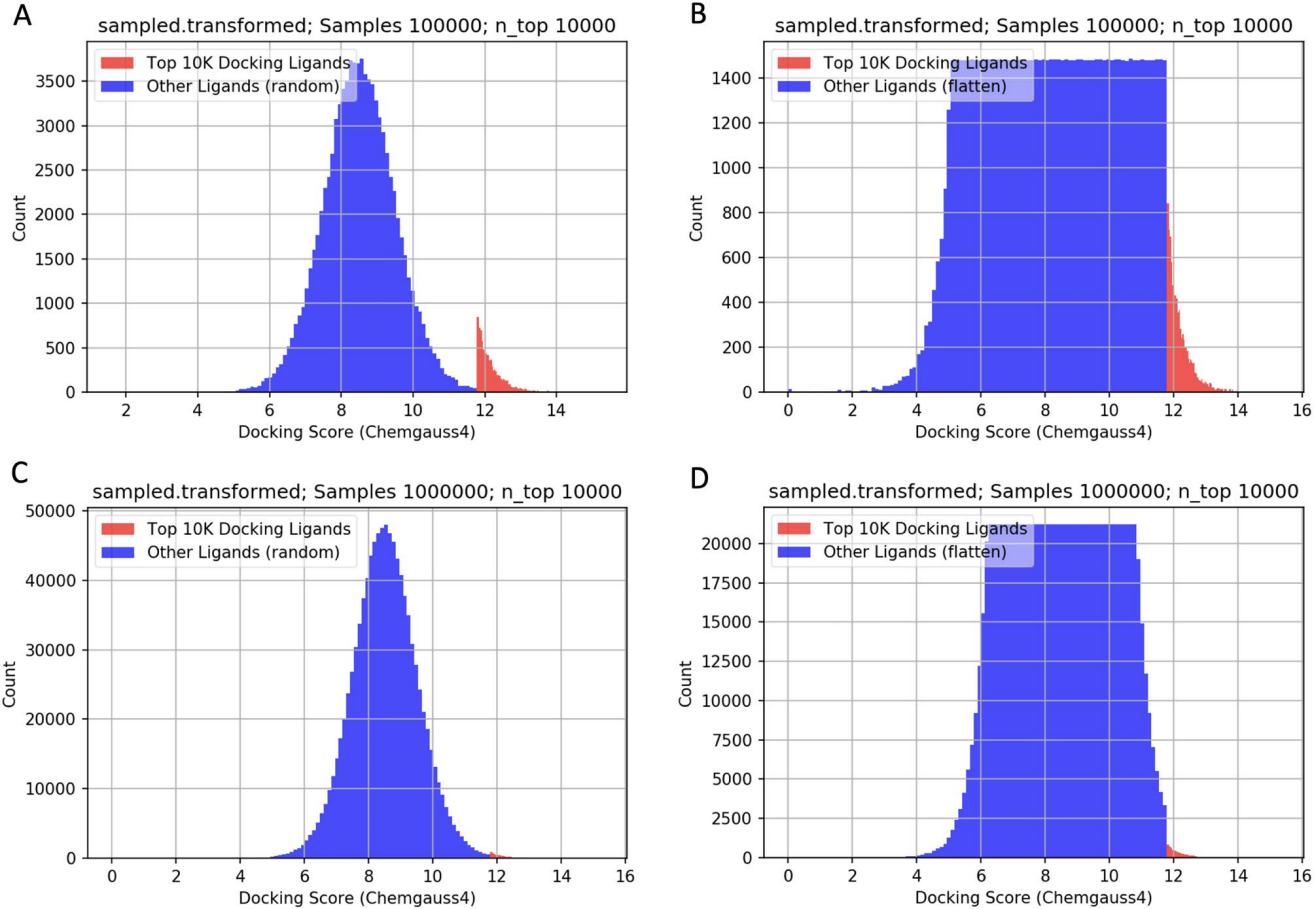
Docking score histograms for each of the four sampling (**a**) 100 K-random, (**b**) 100 K-flatten, (**c**) 1 M-random and (**d**) 1 M-flatten approaches used to generate a subset by sampling the full dataset of available scores (approximately six million samples).

Drawing samples at random preserves the original normal-shaped distribution (thus, the name Random). Alternatively, for a more balanced dataset, we sample scores with an alternative algorithm to create a roughly flattened, uniform-like distribution. To include the highly significant, top score samples, we retain the top ten thousand binding ligands. Figure 3 shows the histograms of the docking scores subset with each of the four sampling scenarios for 3CL-M_pro_. The top ten thousand binding ligands are indicated in red. Note that the distribution of the full dataset can be roughly modeled as a normal distribution, as shown in Fig. 2.

**Table 2:** Impact of including Mordred 3-D descriptors in the training data for the different sampling strategies.

Model	Epoch	Val loss	Val MAE	Val ${r^2}$
1613 features				
V5.1-100 K-flatten-2	337	0.80	0.66	0.71
V5.1-100 K-random-2	336	0.80	0.66	0.71
V5.1-1 M-flatten-2	484	0.60	0.59	0.81
V5.1-1 M-random-2	455	0.49	0.52	0.68
1826 features				
V5.1-100 K-flatten-2	313	0.97	0.74	0.85
V5.1-100 K-random-2	330	0.81	0.67	0.71
V5.1-1 M-flatten-2	462	0.60	0.59	0.81
V5.1-1 M-random-2	456	0.52	0.54	0.67

When examining the impact of including the Mordred 3-D descriptors in the feature set, we average the validation loss, validation MAE, and validation $r^2$ across the 31 models as we are interested in the aggregate performance of the models across the 31 receptors. Our analysis of the inclusion of the Mordred 3D descriptors is presented (Table 2). Our results show no significant advantage to including the 3D descriptors. The results show small improvements in the validation loss across all training data frames when using only the 2D descriptors. The results are mixed when considering validation $r^2$, with two smaller data frames performing slightly better and the two larger data frames performing marginally worse. While we do not consider the differences in most cases to be significant, we demonstrate that adding the extra training parameters in the form of 3D descriptors does not improve the training performance of the model.

When examining the impact of both the training set size (1M or 100K) and sample selection from either a random distribution or flattened distribution, we average the validation loss, validation MAE, and validation $r^2$ for each trained receptor model that represents one of the thirty one different protein pockets. Table 2 shows the differences between the means. A negative value for the validation loss and validation MAE differences would indicate 1M samples achieved a higher quality model, and a positive value for the validation $r^2$ difference would indicate 1M samples achieved a higher quality model. The results indicate that 1M samples from a flattened distribution perform better than 100K samples for all three metrics, whereas 1M samples from a random distribution achieved better metrics for the validation loss and MAE. However, the 1M samples from a random distribution had a lower validation $r^2$.

To better understand the differences between the 1 M data sets, the Pearson correlation coefficient was calculated between predicted and the observed values from the validation set for each pocket model. In the case of the v5.1-1 M samples, the validation set had 200,000 samples. The mean of the PCC across the set of pocket models was calculated for each 1 M data set and the V5.1-1 M-random is 0.853 and the V5.1-1 M-flatten is 0.914.

### Learning curve analysis

To further explore the optimal sample size, we generated learning curves for the 3CLPro receptor model and assume 3CLPro will be indicative of other receptors. Using the entire dataset, which contains approximately 6 M samples, imposes a significant computational burden for training a deep neural network model for each receptor and performing HP tuning. Regardless of the learning algorithm, supervised learning models are expected to improve generalization performance with increasing high-quality labeled data. However, the rate of model improvement achieved by adding more samples diminishes at specific sample sizes. The trajectory of generalization performance as a function training set size can be estimated using empirical learning curves.

The range at which the learning curve starts to converge indicates the sample size where the model begins to exhaust its learning capacity. Figure 2 shows the learning curve where the mean absolute error of predictions is plotted versus the training set size. The curve starts to converge at approximately 1 M samples, implying that increasing sample size beyond this range is not expected to improve predictions.

**Figure 4:**
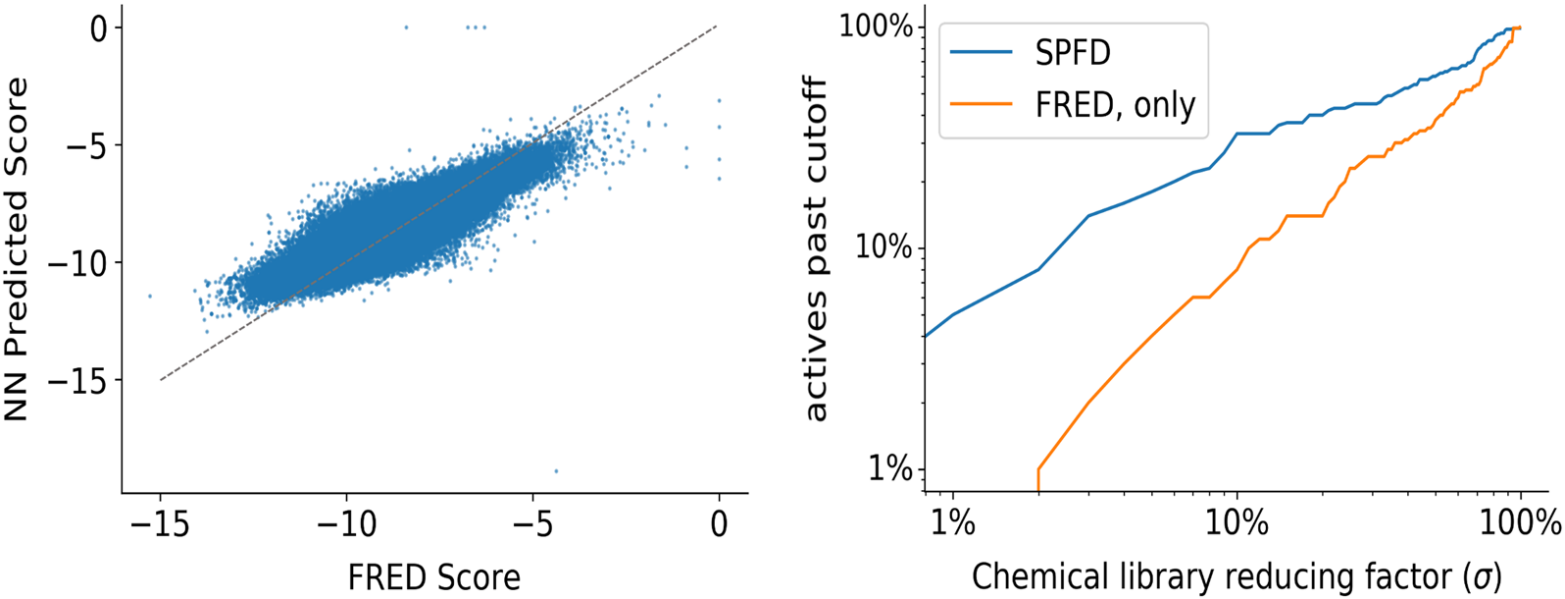
(left) Scatter plot illustrating correlation between the predicted scores and the FRED scores (for 3CL-main protease on a 100,000 random subset of orderable MCule molecules). (right) Detection of active compounds from NCATS (AC50$\le 10 \upmu$M) with SPFD (predicted with NN) and FRED (docking). SPFD detects all active compounds which FRED detects for 3CL-main protease and therefore is a faster alternative to regular docking without loss of active detection. This indicates the differences between the predictions and the actual FRED scores lean towards detecting actives.

### Model accuracy

FRED docking scores correlated (0.825) with the neural network predictions (see Fig. 4). Furthermore, the variation between the NN and the actual FRED scores did not worsen the detection of active molecules. We utilized molecules from a set of 3CL-main protease screening data from National Center for Advancing Translational Sciences open data portal^63^. Molecules from this dataset with an AC50 of 10 μM or less were considered active. Based on a filter cut-off, the NN was able to detect as many active compounds as FRED would (see Fig. 4).

The observations of the data frame comparisons and learning curves show that the 1613 MOrdred 2D descriptors performed better without the inclusion of the 3D based descriptors (in total, 1826 features) in most cases. The 1M data frames performed better than the 100K data frames in most cases. The mean $r^2$ (0.825) of the 1M-flatten was higher than that of the 1M-random data frame (0.721).

**Figure 5:**
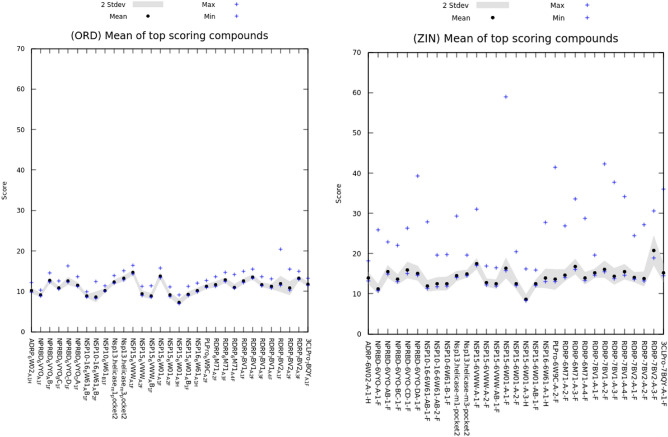
Comparison of the 31 receptor models with the 2000 best scoring compounds from ORD and ORZ.

### Inference across 3.8 billion compounds

We divided the 4 billion compounds into 4 input data sets to enable better utilization of resources. ENA, G13, ZIN, OTH. We also constructed a set of compounds from the MCULE data set that could be easily purchased (organic synthesis already done). The MCULE subset was named ORD. The inferencing rate was approximately 50,000 samples per second per GPU, and all 6 GPUs per summit node were used.

We analyzed the results for each receptor by selecting the top 2000 scoring compounds, and computing mean, standard deviation, maximum, and minimum values. We present two examples of these results in Fig. 5. Interestingly, the range represented by the maximum and minimum predicted scores for the best 2000 scoring compounds is remarkably different between these two. In fact, ZIN was representative of the others (G13, ENA, and OTH). One working hypothesis is that the compounds in ORD are synthesizable, whereas compounds in the other sets are not necessarily synthesizable as these are virtual combinatorial libraries.

**Figure 6:**
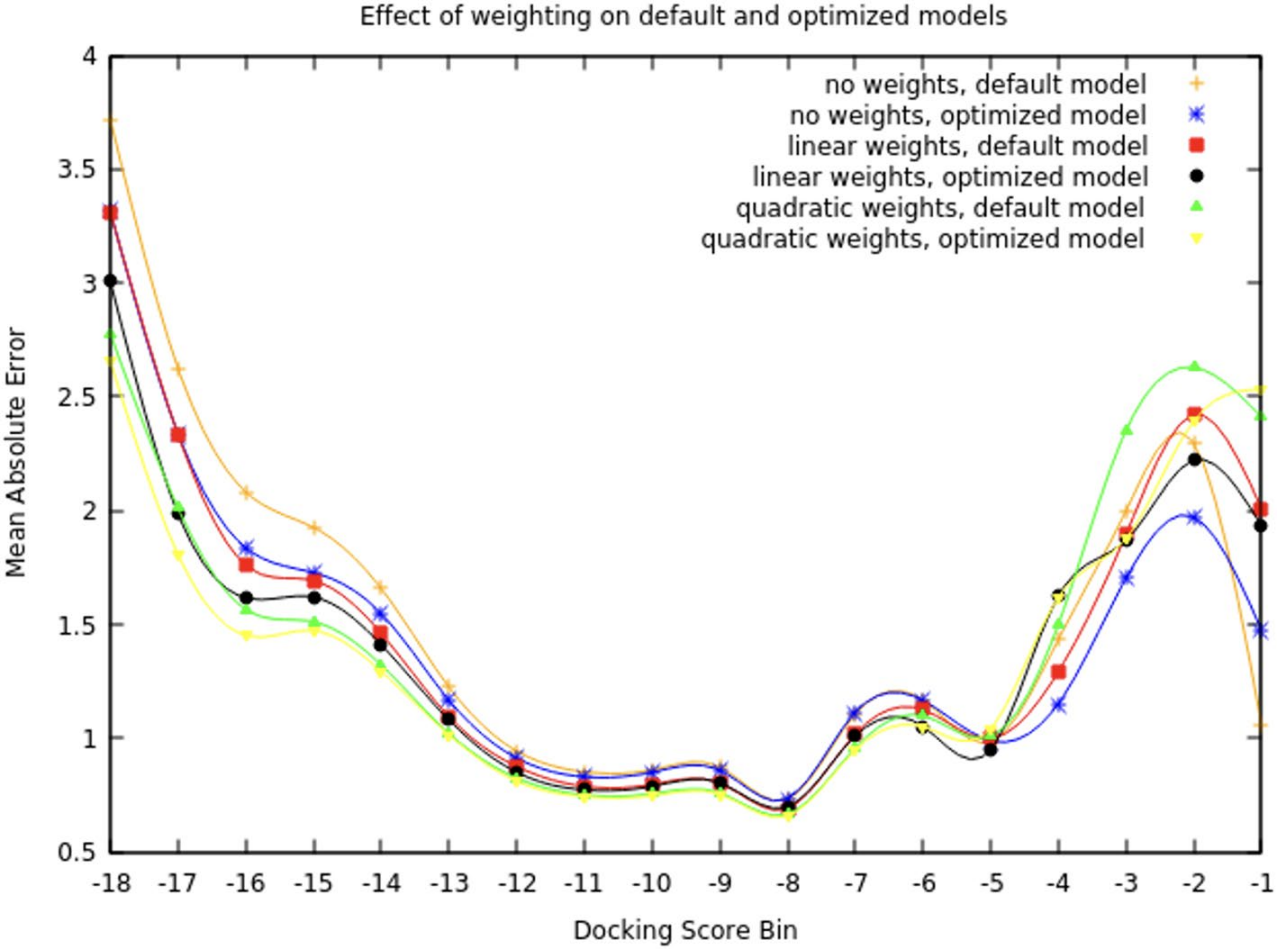
(left) Effects of sample weighting strategies on the default and optimized model. The docking score bins represent buckets where scores fall into and the *y*-axis refers to the mean absolute error (MAE) of a model when using it to predict the docking scores. The different lines represent different optimization strategies between models.

### Model hyperparameter optimization

The CANDLE framework was subsequently used to tune the deep neural network for future training and screening activities^64^. The CANDLE compliant deep neural network was tuned in two phases. The first involved using two CANDLE hyperparameter optimization workflows—mrlMBO and GA. Each differs in the underlying ML techniques used to optimize the hyperparameters. The second phase involved implementing and testing new sample weighting strategies in an attempt to weight the samples at the good end of the distribution more heavily during training. Results of the GA and mlrMBO workflows produced a model architecture that had a 6.6% decrease in the validation mean absolute error and a 2.8% increase in the validation R-squared metrics.

Efforts to decrease the error in the good tail of the distribution (where the docking scores are best) focused on adding sample weights to the model while training. We investigated linear and quadratic weighting strategies. We applied the weighting strategies to both the default model as well as the hyperparameter optimized model. The linear strategy weights the sample proportionally with the docking score, while the quadratic scales with the square of the docking score. These strategies are generic in that they can be applied to basically any training target value. To analyze the impact of the weighting strategies, we computed the mean absolute error on bins of predicted scores with a bin interval of one. These results are presented in Fig. 6.

**Figure 7:**
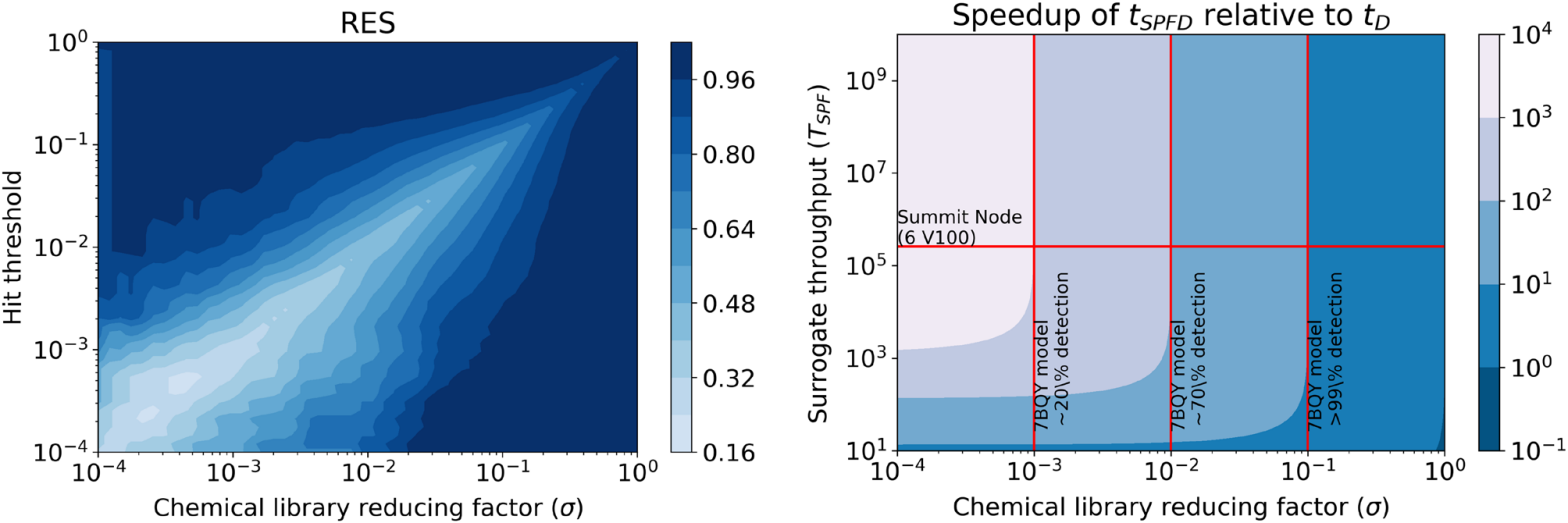
(left) Regression enrichment surface $(n=200,000)$ based on the surrogate model for 7BQY^65^. The *x*-axis represents $\sigma$ which determines the level of filtering the model is used for (i.e., after predicting over the whole library, what percentage of compounds then used in the next stage docking). The *y*-axis is the threshold for determining if a compound is a hit or not. The point $(10^{-1},10^{-3})$ is shaded with 100% detection. This implies the model over a test set can filter out 90% of compounds without ever missing a compound with a score in the $10^{-3}$ percentile. In concrete numbers, we can screen 200,000 compounds with the model, take the top 20,000 based on those inference scores, and dock them. The result is running only 20,000 docking calculations, but those would contain near 100% of the top 200 compounds (as if one docked the entire dataset). (right) Based on equation (1) we compute the relative speedup using surrogate models over traditional workflows with fixed parameters library size (1 billion compounds) and $T_\text {D}=1.37\frac{\text {samples}}{\text {seconds node}}$. The horizontal line indicates where current GPU, surrogate model, throughput is, $T_\text {SPF}$, and the vertical lines correspond to the RES plot values for hit threshold equal to $10^{-3}$. The right-most vertical line implies a VLS campaign with surrogate models where the surrogate GPU-based model can with accuracy $>99\%$ detect the top 10% from the bottom 90% implying a 10 × speedup over traditional methods. By adding surrogate models as a pre-filter to docking, scientists can dock 10x more in the same amount of time with little detectable loss.

## Discussion

Utilizing SPFD we report a 10 × speedup to traditional docking with little to no loss of accuracy or methodical changes needed besides addition of ML-models as a prefilter. Our model detects 99.9% of the high scoring FRED compounds when filtering the dataset at 10%. We show that for a set of active 3CL-main protease compounds that SPFD would not miss any actives that the alternative FRED docking would identify. We see these results as conceptually tying together the model application (hit threshold and adversity to detection loss, choice of $\sigma$) with more traditional analysis such as performance characteristics and model performance evaluation. Furthermore, our data release consists of a square matrix of training data for further study on surrogate regressor models for accelerating docking studies.

To summarize the finding from the various comparisons in the results, we recommend utilizing an initial sample size of 1 M as there was an observed accuracy increase using 1 M initial samples over 100 k; however, using extended features show the possibility of similar performance with only 100 k initial samples if the training data is sampled uniformly from docking bins. Using smaller initial sizes, if possible, decreases the overall training time and required molecular docking runs which increases the overall efficiency of the systems. Smaller initial sample sizes were not tested but will be in future studies. We further recommend uniform sampling the initial data to balance the respective docking scores to the best ability (see Fig. 3). This increased the $r^2$ score by nearly 15% in comparison to randomly sampling the initial training set (from 0.7 to 0.8). We observed significant improvement in the regression correlation (0.71 to 0.85) utilizing the larger feature set of molecular descriptors (1826 compared to 1613). The larger feature set includes 213 extra descriptors which pertain to 3D kernels (though they do not utilize any 3D structure). We recommend utilizing a quadratic weighting scheme as it decreased MAE the most towards the best docking scores, and shows insignificant difference on the least well scoring side of scores (which is less likely to be an error as a bad docking score plus or minus a few points is still bad).

This paper asks how can standard docking protocols be accelerated for large billion scale screening? Our timing analysis implies that to achieve a speedup beyond a single order computation does not need to be faster. Rather, the limiting factor to accelerating the workflow is a need for more accurate regressor models. Our analysis, outlined in Fig. 7, highlights the choice of prefilter threshold as the limiting factor for seeing orders of magnitude speedup. In particular, focusing on speedups which show *no loss* of detection, model accuracy must be pressed forward as there is no path to accelerating traditional docking workflows without more accurate surrogate models. Given out of box modeling technique can speed up virtual screening 10 × with no loss of detection power for a reasonable hit labeling strategy (top 0.1%), we believe the community is not far from 100 × or even 1000 ×. The way to get there is to boost our model accuracies or develop techniques to recover hits in lossy SPFD regimes (such as not improving model performance but decreasing $\sigma$ to $10^{-2}$ and applying another technique to recover the 30% loss of detection power). This benchmark is important, as successful early drug discovery efforts are essential to rapidly finding drugs to emerging novel targets. Improvements in this benchmark will lead to orders of magnitude improvement in drug discovery throughput.

We discuss the relative speedup of utilizing a pre-filter surrogate model for docking campaigns against a traditional docking campaign. We define two workflows for performing protein-ligand docking over a library of compounds: D (traditional docking, no surrogate-prefilter) and SPFD (surrogate-prefilter then dock). We construct timing models of both of these workflows to understand the relationship between computational accuracy, computational performance (time and throughput), and pre-filter hyperparameter ($\sigma$). We distinguish between the surrogate model’s accuracy, which pertains to how well the surrogate model fits the data, and workflow accuracy, which pertains to how well the results of the whole SPFD workflow compares to the results of just traditional docking workflow.

As discussed in the methods section, the choice of pre-filter hyperparameter, $\sigma$, is a decision about workflow accuracy for detecting top leads. Model accuracy influences the workflow accuracy, but the workflow accuracy can be adjusted with respect to a fixed model accuracy (see Fig. 7 where the vertical lines each correspond to the same underlying model with fixed accuracy but differentiate the overall workflow accuracy with respect to traditional docking). Therefore we can interpret $\sigma$ as a trade-off between the workflow throughput and workflow accuracy. For example, $\sigma =1$ is always 100% workflow accurate since traditional docking is run on the whole library when $\sigma =1$, but $\sigma =1$ is even slower than traditional docking as it implies docking the whole library as well as utilizing the surrogate model. We can determine the overall workflow accuracy with the model by looking at the RES plot, which of course has as a factor the performance characteristics of the surrogate model. Given a particular model’s accuracy versus performance characteristics, different levels of pre-filtering ($\sigma$), correlate to different tolerances to detecting top-scoring compounds.

For the following analysis, we fix node types for simplicity. Let *L* be the number of compounds in a virtual library to screen. Assume the traditional protein-ligand docking software has a throughput $T_{\text {D}}$ in units $\frac{\text {samples}}{\text {(seconds)(node)}}$, and the surrogate models have a throughput $T_{\text {SPF}}$. Let $t_\text {D}$ and $t_\text {SPFD}$ be the wall-clock time of the two workflows. The time of the traditional workflow, $t_\text {D}$, and the time of the surrogate prefilter then dock workflow, $t_\text {SPFD}$, are

1 $$\begin{aligned} t_\text {D}=\frac{L}{T_\text {D}}\quad \text {and}\quad t_\text {SPFD} = \frac{\sigma L}{T_\text {D}} + \frac{L}{T_\text {SPF}}. \end{aligned}$$

Notice, that $t_\text {SPFD}$ is simply the sum of the time of running the surrogate model over the library, $L/T_\text {SPF}$, and the time of traditional docking the highest scoring $\sigma L$ compounds. The time to train the deep learning model is excluded as it is constant time (assuming 100 k docking scores are used to bootstrap the model). Furthermore, the training time of our proposed neural network is roughly two to three hours on a single NVIDIA A100 GPU which is rather small compared to the run-time on hundreds of supercomputer nodes for large-scale docking studies.

2 $$\begin{aligned} \text {Speedup} = \frac{T_\text {SPF}}{T_\text {D} + \sigma T_\text {SPF}} \end{aligned}$$

This implies that the ideal speedup of our workflow is directly dependent on the throughput of both the docking calculation, surrogate model, and the parameter $\sigma$. $\sigma$ is indirectly dependent on the model accuracy. If the surrogate model was completely inaccurate, even though $\sigma =10^{-3}$ implies a 1000 × speed up, no hits would be detected. If one wants to maximize workflow accuracy, that is not miss any high scoring compounds compared to traditional docking, then they must supply a threshold for hits (corresponding to the *y*-axis of RES). Suppose this threshold is $y_\text {thres}=10^{-3}$. If they wanted to maximize not missing any compounds, they should set $\sigma$ to $10^{-1}$ based on this model’s RES plot since that is the smallest value of $\sigma$ such that the detection accuracy of the surrogate model is near 100%. But, it is not always the case downstream tasks require 100% detection—hence $\sigma$ is a true hyperparameter.

We infer $T_{\text {SPF}}$ on a Summit (Oak Ridge Leadership Computing Facility) and on an A100 ThetaGPU node (Argonne Leader Computing Facility). Both tests were using 64 nodes, 6 GPUs per node, but the throughput was computed per GPU. We found the V100 summit node was capable of 258.0 K$\frac{\text {samples}}{\text {(s)(node)}}$ while the A100 nodes were 713.4 K $\frac{\text {samples}}{\text {(s)(node)}}$. We infer $T_\text {D}$ as 1.37 $\frac{\text {samples}}{\text {(s)(node)}}$ based on a CPU docking run over 4000 summit nodes with 90% CPU utilization from^6^. Thus, we can compute the speedup based on a Summit node head-to-head comparing setting $T_\text {SPF}$ to 258.0 K and $T_\text {D}$ to 1.37 in Eq. (1), resulting in a speedup of 10 × for $\sigma =0.1$. Based on the RES analysis in Figure 7, $\sigma$ of 0.1 corresponds to a model accuracy of near 100% for filtering high scoring leads ($>1$% of library). If one is willing to trade-off some loss of detection, say 70% detection of high scoring leads, then the speedup is 100 ×. The extreme case, a choice of $\sigma =10^{-3}$, implies a speedup 1000 × but means only roughly 20% of the top scoring leads may appear at the end.

Therefore, our analysis of SPFD implies that speedups are essentially determined by $\sigma$ while $T_\text {SPF}$ does not have as large of an effect (this is based on how fast ML inference currently is). As a hyperparameter, $\sigma$ is dependent on the workflow’s context and, in particular, what the researchers are after for that SBVS campaign. We can say, though, at least informally, model accuracy and $\sigma$ are highly related. The more accurate the models are, the better the RES plot gets as one is willing to trust the ML model for filtering the best compounds from the rest. In Fig. 7, the *x*-axis of both plots are similar. The accuracy of a particular $\sigma$ is found by setting one’s level of desired detection, the *y*-axis of the RES plot, and then checking the $(\sigma , y)$ point to see how accurate the model is there. The choice of $\sigma$ is subjective based on how accurate one needs the model for their *y*-axis threshold for hits. We focus mainly on the case of no loss of detection, which means $\sigma =0.1$ for our particular trained models. In order to focus on the theoretical model of relating computational accuracy, confidence (again, in a colloquial sense), and computational performance, we simplify over a richer model of performance analysis assuming uniformity of task timing and perfect scaling. Furthermore, we chose a head-to-head comparison of a particular node type’s CPU performance to GPU performance. At the same time, we could have compared the best non-surrogate model workflow times to the best surrogate model workflow times.

## Conclusion

We demonstrate an accelerated protein-ligand docking workflow called surrogate model prefiltering then dock (SPFD), which is at least 10 × faster than traditional docking with nearly zero loss of detection power. We utilize neural network models to learn a surrogate mode to the CPU-bound protein-ligand docking code. The surrogate model has a throughput over six orders of magnitude faster than the standard docking protocol. By combining these workflows, utilizing the surrogate model as a prefilter, we can gain a 10 × speedup over traditional docking software without losing any detection ability (for hits defined as the best scoring 0.1% of a compound library). We utilize regression enrichment surfaces to perform this analysis. The regression enrichment surface plot is more illustrative than the typical accuracy metrics reported from deep learning practices. Figure 7 showcases our initial models at this benchmark show a 10 × speedup without loss of detection (or 100 × speedup with 70% detection). We released over 200 million 3D pose structures and associated docking scores across the SARS-CoV-2 protemome This 10 × speedup means if a current campaign takes one day to run on library size *L*, one can screen ten times as many compounds in the same amount of time without missing leads. Given the potential for 100 × or even 1000 × speedup for docking campaigns, we hope to advance the ability of surrogate models to filter at finer levels of discrimination accurately.

## Supplementary Information

Supplementary Information.

## Data Availability

All data is available online at https://github.com/2019-ncovgroup/HTDockingDataInstructions as well as https://doi.org/10.26311/BFKY-EX6P.
