# Supplementary material for: AI-accelerated protein-ligand docking for SARS-CoV-2 is 100-fold faster with no significant change in detection
Source: Sci Rep. 2023 Feb 6;13:2105. doi: 10.1038/s41598-023-28785-9 (PMC9901402; doi:10.1038/s41598-023-28785-9)
Supplement: Supplementary file 1 — Supplementary Information. [file 41598_2023_28785_MOESM1_ESM.pdf]

Supplementary Information

0.1 ML Dataframe Description

This dataset contains ML data-frame designed for systematic comparison across models using different features. It includes the top promising 712199 molecules selected through pipeline from billion molecules from various data-sets in section 0.1.1 and 24 receptors in section 0.2 for 2019 nCoV. The ML dataframe contains features including MACCS keys (see section 0.1.4), ECFP2 (see section 0.1.5), ECFP4 (see section 0.1.5), ECFP6 (see section 0.1.5), Descriptors (see section 0.1.6). We dropped all the rows that had a NAN and made alignment across all the files. So all the files have the same molecular order with various complete features.

0.1.1 Datasets

The molecules in the ML dataframe were selected from the datasets 3 below.

Dataset Name
ZINC15[20]
SureChEMBL dataset of molecules from patents[66]
Synthetically Accessible Virtual Inventory (SAVI)[67]
Repurposing-related drug/tool compounds[68]
QM9 subset of GDB-17[69]
PubChem[70]
MCULE compound database
Molecular Sets (MOSES)[71]
Harvard Organic Photovoltaic Dataset[72]
GDB-17-Set up to 17 atom extension of GDB-13[73]
GDB-13 small organic molecules up to 13 atoms[74]
CureFFI FDA-approved drugs and CNS drugs
Enamine REAL Database[21]
eMolecules
DrugBank plus Enamine Hit Locator Library 2018
Diverse REAL drug-like subset of ENA
DUDE database of useful decoys[54]
DrugCentral Online Drug Compendium[75]
Drugbank[75]
The Binding Database[76]

Table 3. The top promising molecules were collected from the above dataset

0.1.2 SMILES

The SMILES (Simplified Molecular Input Line Entry System)[77] converts the chemical species into a linear text format. By using SMILES, we could convert it back into two or three dimensional representation of molecules.

0.1.3 Canonical SMILES

A single molecular could be represented by different SMILES strings. Canonical SMILES give a unique SMILES string for a single molecular.

0.1.4 MACCS keys

The MACCS (Molecular Access System) keys[50] are one of the fingerprints and structural keys. MACCS keys has 166-bit 2D structure fingerprints. They are widely used for molecular similarity measurement.

0.1.5 ECFP2, ECFP4, ECFP6

ECFP2, ECFP4 and ECFP6 are Extended-connectivity fingerprints of diameter 2, 4 and 6 respectively, which are a topological fingerprints for molecular characterization. They could be used for substructure and similarity searching. In the ML dataframes, we encode the ECFP2, ECFP4 and ECFP6 by the length of 512 and 2048 respectively.

0.1.6 Molecular Descriptors

Molecular descriptors[78] described the properties of a molecular in different aspects. They are theoretically-derived or experimentally measured properties of a molecular. We used Mordred[60] to generate the molecular descriptors. We collected 1613 descriptors for each molecular.

0.2 Receptors

Our dataframe includes 24 receptors in Table 4.

Receptor \ coordinates of the bounding box	x min	x max	y min	y max	z min	z max
3CLPRO_1	-16.24	-1.90	-9.85	9.81	-30.77	-13.77
3CLPRO_2	0.09	10.42	-25.53	-6.86	-14.90	-1.57
3CLPRO_3	-15.29	-1.62	14.42	27.42	-12.99	-2.65
ADRP_ADPR_A	-3.16	14.17	-15.16	2.84	-33.81	-12.48
COV_RDB_AB	-21.69	-2.36	-3.60	11.74	0.11	15.45
COV_RDB_A_1	-29.43	-12.10	-16.82	-0.48	-20.01	-1.34
COV_RDB_BC	-15.99	0.01	19.16	31.50	14.29	26.29
COV_RDB_CD	-19.38	-2.05	27.96	43.29	-2.15	12.85
COV_RDB_DA	-15.79	1.21	-0.55	19.79	-17.43	-1.43
DNMT1_CHAINA	13.69	34.02	-37.77	-13.43	21.95	53.62
DNMT3A_CHAINA	48.53	67.86	23.81	40.14	-40.97	-14.97
MPRO-X0104	2.02	16.36	-9.06	11.27	15.21	29.21
MPRO-X0107	-0.25	15.08	-8.06	7.94	14.87	27.87
MPRO-X0161	2.77	17.44	-7.51	10.49	15.72	27.39
MPRO-X0305	-0.80	16.53	-9.04	9.29	14.19	32.52
NSP15_1_6VWW	-83.85	-60.19	18.81	37.81	-35.20	-17.87
NSP15_1_6W01	-73.68	-52.68	38.94	59.60	18.46	37.80
NSP15_2_6VWW	-73.05	-60.05	-3.28	19.39	-39.42	-21.08
NSP15_2_6W01	-51.50	-26.16	49.02	58.02	20.89	39.89
NSP15_3_6W01	-72.41	-58.41	66.08	78.08	21.81	35.81
NSUN2	10.11	26.44	-17.02	-0.36	46.01	57.68
NSUN6	56.52	71.18	-13.75	13.25	17.79	42.13
PLPRO_1	-22.60	-3.27	37.20	52.53	-48.62	-33.62
PLPRO_2	-4.39	13.28	32.70	48.37	-19.76	-6.76

Table 4. The receptors in the ML dataframe.

0.3 Models Details

For baseline models, we used a fully connected deep neural network with hidden layers and dropout layers in between. For the descriptor baseline, we selected the top 512 features from 1613 and used four hidden layers with neuron counts [512, 250, 125, 30, 1]. For fingerprint, we used ECFP4 with 512 features as a baseline and set the neuron counts of five hidden layers as [512, 250, 125, 60, 30, 1]. We set the dropout rate to 0.1. The batch size (number of samples for gradient descent) was set to 50. The epoch was set to 100. We used mean square error (MSE) as the loss function and stochastic gradient descent (SGD) as the optimizer. We set the optimizer with an initial learning rate of 0.00001 and momentum set to 0.9. The implementation was python using PyTorch.

0.4 Baselines

We generated the baselines in Table 5 by using models in section 0.3. The simple baselines were created to give a quick idea about how well your models performs.

0.4.1 Descriptor and Fingerprint

target_name	loss<br>Descriptor, Fingerprint	correlation<br>Descriptor, Fingerprint	r2-score<br>Descriptor, Fingerprint	CI<br>Descriptor, Fingerprint
MPRO-X0161	0.966, 0.997	0.640, 0.622	0.406, 0.387	0.730, 0.721
NSP15_2_6VWW	0.446, 0.478	0.868, 0.858	0.753, 0.735	0.841, 0.835
ADRP_ADPR_A	1.156, 1.191	0.691, 0.678	0.475, 0.459	0.738, 0.731
NSP15_1_6W01	0.631, 0.629	0.780, 0.780	0.606, 0.608	0.786, 0.786
3CLPRO_3	0.486, 0.529	0.809, 0.790	0.654, 0.624	0.799, 0.790
COV_RDB_CD	0.569, 0.601	0.752, 0.735	0.565, 0.540	0.769, 0.761
COV_RDB_DA	0.623, 0.668	0.811, 0.794	0.655, 0.631	0.803, 0.795
COV_RDB_BC	0.694, 0.781	0.856, 0.836	0.733, 0.699	0.829, 0.818
NSP15_3_6W01	0.907, 0.956	0.745, 0.728	0.554, 0.530	0.773, 0.765
DNMT1_CHAINA	0.499, 0.611	0.904, 0.882	0.817, 0.775	0.854, 0.840
3CLPRO_1	0.464, 0.503	0.813, 0.795	0.660, 0.631	0.804, 0.794
COV_RDB_A_1	0.404, 0.416	0.800, 0.793	0.639, 0.628	0.797, 0.793
MPRO-X0104	1.050, 1.060	0.720, 0.714	0.515, 0.510	0.760, 0.755
PLPRO_1	0.850, 0.879	0.844, 0.837	0.710, 0.700	0.824, 0.820
NSUN2	0.408, 0.452	0.872, 0.857	0.760, 0.734	0.842, 0.833
DNMT3A_CHAINA	0.657, 0.700	0.810, 0.797	0.656, 0.634	0.797, 0.791
NSUN6	0.478, 0.497	0.820, 0.812	0.671, 0.657	0.806, 0.800
NSP15_2_6W01	0.431, 0.629	0.865, 0.780	0.748, 0.608	0.837, 0.786
3CLPRO_2	0.427, 0.448	0.821, 0.810	0.673, 0.657	0.811, 0.805
MPRO-X0107	1.195, 1.161	0.732, 0.740	0.534, 0.547	0.764, 0.766
PLPRO_2	0.464, 0.503	0.796, 0.776	0.633, 0.602	0.792, 0.783
NSP15_1_6VWW	0.641, 0.645	0.784, 0.782	0.613, 0.611	0.788, 0.787
MPRO-X0305	0.996, 1.054	0.753, 0.735	0.565, 0.540	0.776, 0.768
COV_RDB_AB	0.589, 0.626	0.745, 0.725	0.553, 0.525	0.764, 0.755

Table 5. baselines for descriptors and fingerprints on various receptors
